# Dual-stage cognitive assessment: a two-stage screening for cognitive impairment in primary care

**DOI:** 10.1186/s12888-023-04883-w

**Published:** 2023-05-25

**Authors:** Liang Cui, Zhen Zhang, Lin Huang, Qinjie Li, Yi-Han Guo, Qi-Hao Guo

**Affiliations:** 1 Department of Gerontology, Shanghai Sixth People’s Hospital, Shanghai Jiao Tong University School of Medicine, Shanghai, 200233 China; 2 Faculty of Medicine, The University of Queensland, Brisbane, Australia

**Keywords:** Mild cognitive impairment, Visual memory, Auditory memory, Primary care, Geriatric assessment

## Abstract

**Background:**

Aging population has led to an increased proportion of older adults and cognitively impaired. We designed a brief and flexible two-stage cognitive screening scale, the Dual-Stage Cognitive Assessment (DuCA), for cognitive screening in primary care settings.

**Method:**

In total, 1,772 community-dwelling participants were recruited, including those with normal cognition (NC, n = 1,008), mild cognitive impairment (MCI, n = 633), and Alzheimer’s disease (AD, n = 131), and administered a neuropsychological test battery and the DuCA. To improve performance, the DuCA combines visual and auditory memory tests for an enhanced memory function test.

**Results:**

The correlation coefficient between DuCA-part 1 and DuCA-total was 0.84 (P < 0.001). The correlation coefficients of DuCA-part 1 with Addenbrooke’s Cognitive Examination III (ACE-III) and Montreal Cognitive Assessment Basic (MoCA-B) were 0.66 (P < 0.001) and 0.85 (P < 0.001), respectively. The correlation coefficients of DuCA-total with ACE-III and MoCA-B were 0.78 (P < 0.001) and 0.83 (P < 0.001), respectively. DuCA-Part 1 showed a similar discrimination ability for MCI from NC (area under curve [AUC] = 0.87, 95%CI 0.848–0.883) as ACE III (AUC = 0.86, 95%CI 0.838–0.874) and MoCA-B (AUC = 0.85, 95%CI 0.830–0.868). DuCA-total had a higher AUC (0.93, 95%CI: 0.917–0.942). At different education levels, the AUC was 0.83–0.84 for DuCA-part 1, and 0.89–0.94 for DuCA-total. DuCA-part 1 and DuCA-total’s ability to discriminate AD from MCI was 0.84 and 0.93, respectively.

**Conclusion:**

DuCA-Part 1 would aid rapid screening and supplemented with the second part for a complete assessment. DuCA is suited for large-scale cognitive screening in primary care, saving time and eliminating the need for extensively training assessors.

**Supplementary Information:**

The online version contains supplementary material available at 10.1186/s12888-023-04883-w.

## Introduction

The accelerated aging of the Chinese population is posing a public health challenge [1]. Currently, approximately 15 million people aged > 60 years have dementia in China [2]. Dementia causes tremendous suffering in patients and substantial economic burden for society. To address this challenge, screening for mild cognitive impairment (MCI) must be prioritized and promoted. Establishing health records of the 260 million older adults in China will help identify patients with cognitive impairment. However, primary care resources in China are inadequate. In China, raters are not considered professionals, and no registration system exists. Therefore, the number of raters with neuropsychological training is insufficient.

There are many approaches to identifying dementia, either from informants [3], such as using the Alzheimer’s disease 8 (AD8) [4] and the Informant Questionnaire on Cognitive Decline in the Elderly (IQCODE) [5] or from daily life observations, such as the Activity of Daily Living (ADL) scale [6]. Cognitive screening measures for dementia such as the Mini-Mental State Examination (MMSE) [7], Montreal Cognitive Assessment (MoCA) [8], and Clock Drawing Test (CDT) [9] are commonly used.

The overall prevalence of MCI in China is estimated to be 15.5% [2]. Large-scale rapid screening of community residents by primary care providers (e.g., family physicians) is a practical approach to improve the diagnosis and management of cognitive impairment in such a huge population. Therefore, appropriate cognitive-screening scales are required. However, the current Chinese versions of these screening scales have limitations for screening MCI. In the Chinese version of these screening scales, memory assessment comprises of only one item. The memory assessment component of Memory and Executive Screening (MES) [10] is the auditory memory of a sentence with 10 key points, while that of the Hong Kong Brief Cognitive Test (HKBC) [11] is auditory memory of four objects. The memory assessment component of the Five-Minute Cognitive Test [12] is visual memory of eight objects. The Geriatric Cognitive Comprehensive Assessment Examiner-Rating Scale (GCCAES) [13] examines three-word auditory memory similar to the MMSE. However, these four screening scales do not fully reflect memory function. Except for the MES, the difficulty of the memory items is lower than that of the MoCA.

The MoCA is one of the most commonly used screening scales; however, it is highly time-consuming and unsuitable for amnestic MCI screening. The MoCA has only five points for memory items (25/30 points on the MoCA are not situational episodic memory items), and some items are not sensitive enough for screening MCI, such as 100 consecutive minus seven and digit spans. Another commonly used scale, the MMSE, has similar issues regarding memory function evaluation.

Therefore, identifying MCI quickly and accurately in the Chinese cultural background requires the development of new, rapid, and practical tools. Hence, we conceived a two-stage process. The first part of the instrument was designed to be very short, taking approximately three minutes to complete, and providing good sensitivity and acceptable specificity. This process was intended to exclude those with good cognitive function and those with poor cognitive function in the community, to save assessor resources. The former does not require further assessment, whereas the latter requires direct referral to a specialized facility for a detailed assessment and specialist examination. It is worth clarifying that cognitive impairment may involve impairment in multiple cognitive domains, and even cognitive impairment due to AD may have an atypical presentation. Our design enhances the detection of memory functions rather than assess the full range of cognitive domains. This was done to allow lay assessors in primary care, usually in the community, to detect cognitive impairment due to typical AD within the shortest possible time. Individuals with suspected cognitive impairment detected using the first part should be assessed using the second part of the test. Therefore, the second part improves the specificity of the entire tool.

A final definitive diagnosis of cognitive impairment can only be made in hospitals or specialized facilities. It requires multiple assessments, biomarker testing, and precise diagnosis by a medical professional based on appropriate diagnostic criteria. Biomarkers such as β-amyloid are becoming increasingly prevalent in the diagnosis of AD [14]. Primary healthcare facilities usually do not have the necessary equipment or capacity to detect biological markers using positron emission tomography or cerebrospinal fluid analysis. If cognitive screening can help identify biomarkers, it can increase the ability for initially determining cognitive impairment pathology in primary care settings. We have added an assessment of prospective memory [15] and metamemory [16], which may be more sensitive or related to β-amyloid pathology, to this new assessment tool. We hope to validate their role in the identification of biomarkers in subsequent studies.

In summary, the huge community population in China needs to be screened for cognitive impairments; however, limited assessment resources are available. Therefore, a flexible, effective, and rapid assessment tool was required. This study aimed to develop a cognitive screening scale, the Dual-Stage Cognitive Assessment (DuCA), for cognitive function screening in primary care. The characteristics of the DuCA may enable the rapid screening of older adults with cognitive impairment in the community. To our knowledge, no similar two-stage screening tool has been used on a large scale in China. A novel assessment tool, such as DuCA, would aid large-scale cognitive screening in primary care.

## Methods

### Participants

The participants were recruited from a cognition clinic and the local community. All participants had to meet the following criteria: (1) native Chinese speakers; (2) no history of stroke, craniocerebral injury, brain tumor, anxiety or depression, or other systemic diseases that affect brain function; and (3) absence of severe hearing or visual impairment, enabling them to complete the neuropsychological test. Individuals with (1) a history of alcohol or drug abuse, psychiatric disorders, epilepsy, head trauma, stroke, or other severe neurological disorders; (2) significant thyroid function abnormalities or syphilis serology; and (3) dementia or MCI caused by known non-AD etiologies such as stroke, Parkinson’s disease, Lewy body dementia, or frontotemporal degeneration were excluded.

### Neuropsychological tests and diagnostic criteria

Basic assessments were conducted for all participants, including the Mini-Mental State Examination (MMSE) [7], Montreal Cognitive Assessment Basic (MoCA-B) [8], Addenbrooke’s Cognitive Examination III (ACE III) [17], Subjective Cognitive Decline Interview (SCD-I) [18], Everyday Cognition (ECOG) [19], Functional Activity Questionnaire (FAQ) [20], and ADL [6].

MCI was diagnosed using the 2011 National Institute on Aging-Alzheimer’s Association (NIA-AA) 2011 criteria [21]. Diagnostic assessments were performed using the following neuropsychological tests: the Auditory Verbal Learning Test (AVLT) [22], Brief Visuospatial Memory Test-Revised (BVMT) [23], Animal Fluency Test (AFT) [24], Boston Naming Test (BNT) [25], Shape Trail Test (STT)-A, and STT-B [26]. An indicator with more than one standard deviation below the age-corrected mean indicates impairment [27].

AD was diagnosed using the NIA-AA 2011 diagnostic criteria for probable AD [28]. Specifically, the diagnostic criteria for AD met the criteria for dementia and had the following characteristics: (1) insidious onset; (2) clear-cut history of worsening of cognition by report or observation; and (3) initial and most prominent cognitive deficits manifested as amnestic presentation or the following non-amnestic presentations: language function impairment, visuospatial function impairment, or executive function impairment [28].

### DuCA

The DuCA was designed as a two-stage assessment of cognitive impairment. In the rapid screening stage, part 1 was used to screen for cognitive function within approximately 3 min. Part 2 was administered to people with suspected cognitive impairment and forms the final total score along with Part 1. Table S1 shows the complete contents of the DuCA. To thoroughly test the performance of the DuCA, all participants underwent the two-part assessment.

The total score for part 1 was 10 points. In Part 1, the items used to calculate the total score were verbal fluency (two points), visual perception (three points), and delayed recall (five points). Initially, the tester orally presented five words to the participants and asked them to remember and recall them immediately. Scores for immediate recall were not included in the total score; this step was intended as a preparation for delayed recall. The verbal fluency item required participants to name as many fruits as possible in 1 min. For visual perception item, the tester presented a picture of 10 objects with overlapping silhouettes and asked the participants to name these objects. For the delayed recall item, participants were asked to name five words that they remembered from the immediate recall item (Table S1 in the Supplement).

The total score for part 2 was 28 points. In Part 2, the items used to calculate the total score were auditory sentence memory (six points), category switching (10 points), and visual memory (12 points). Initially, the tester presented pictures of 12 objects and asked the participants to name them. After observing these pictures for 30 s, the participants were asked to perform an immediate recall. After observing the pictures again for 1 min, a second immediate recall was performed. Points for image naming and the first immediate recall were not included in the total score. For the auditory sentence memory item, the testers orally stated a sentence with three names and four address elements. Participants were then asked to repeat the sentence, and the correct elements were recorded. For the category-switching test item, the participants were requested to orally generate objects in the order of alternating animals and fruits within 60 s, such as dogs, apples, horses, oranges, mice, and bananas. Next, delayed visual memory recall was performed. The participants were asked to recall the objects in the 12 pictures. The visual memory score was the total score of the second immediate recall and delayed free recall, divided by two.

The total score obtained by adding these two scores was 38. The DuCA comprised additional items on prospective memory and metamemory. These items do not count toward the total score, but provided additional information. We plan to cover prospective memory and metamemory in future studies.

### Statistics

Data analysis was performed using SPSS (SPSS Inc., Chicago, IL, USA) and Jamovi (https://www.jamovi.org/) software. Participants with missing diagnostic neuropsychological evaluations were excluded, as previously described. Potential confounders, including sex, age, and years of education, were compared between groups. Descriptive statistics for the DuCA used sex as the stratification factor. Categorical variables were presented using percentages, and quantitative data were presented as mean ± standard deviation (SD). Categorical data were analyzed using the Chi-square test. Quantitative data were analyzed using the t-test between two groups, effect size was reported, and multiple group comparisons were performed using one-way analysis of variance (One-way ANOVA). The Games-Howell test was used for post-hoc analyses. Correlations between neuropsychological test scores were estimated using Pearson’s correlation coefficients. Logistic regression analysis and the area under the curve (AUC) for receiver operating characteristic (ROC) analysis were used to describe the discriminative capabilities of the assessments. In the logistic regression analysis, years of education was used as a stratifying factor.

## Results

### Demography and general neuropsychological performance

**Fig. 1:**
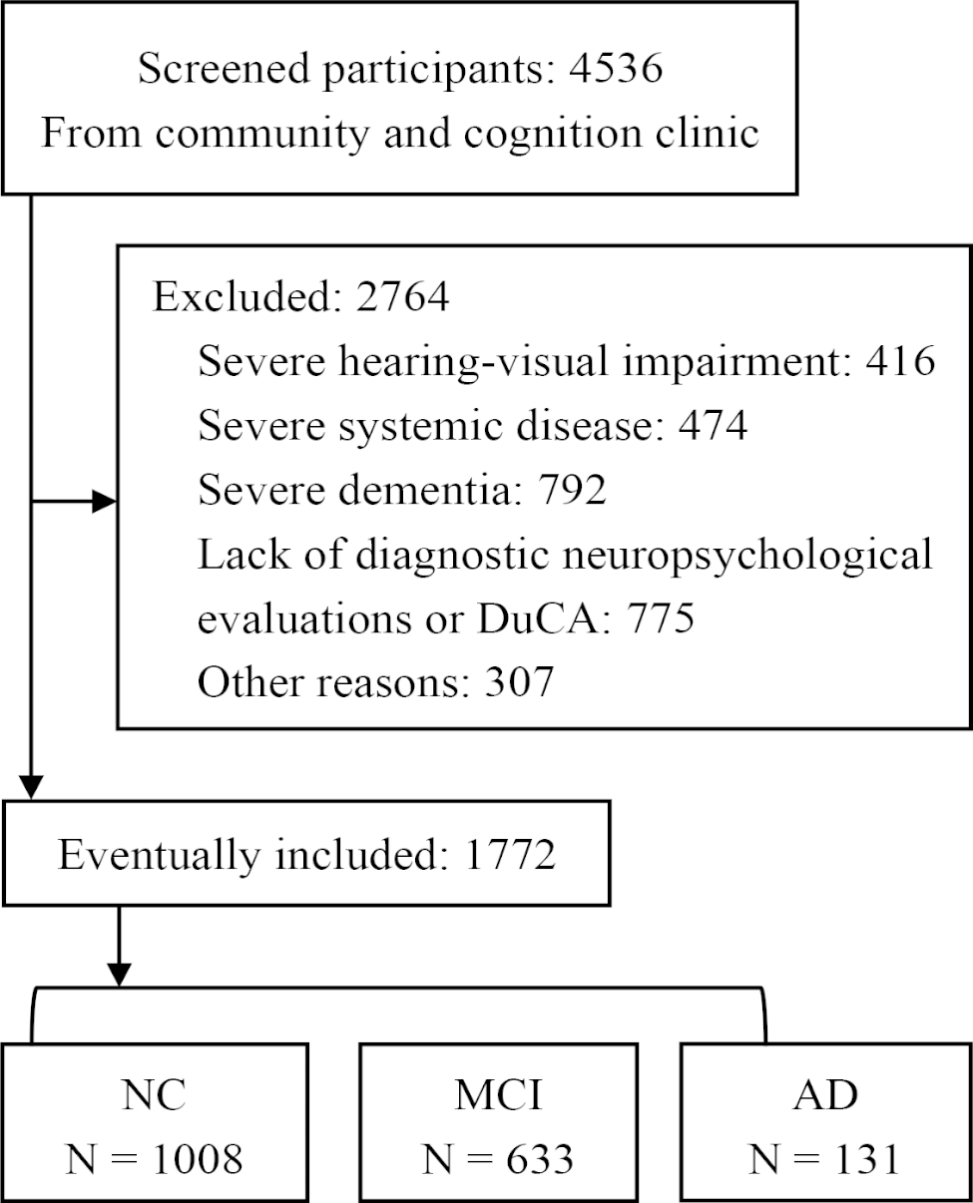
Study flow chart

In total, 4,536 participants were screened between April 2019 and July 2022. Among these, 1,989 participants were excluded because they did not meet the inclusion criteria or met the exclusion criteria; further, 775 participants were excluded because of failure to complete the required neuropsychological test battery or DuCA. Eventually, 1,772 participants were included in the analysis (Fig. 1). The normal cognition (NC) group included 1,008 participants (359 men, aged 65.12 ± 6.99 years, education 12.21 ± 3.25 years). The MCI group included 633 participants (206 men, aged 66.14 ± 6.78 years, education 11.03 ± 3.21 years). The AD group included 131 participants (51 men, aged 71.99 ± 6.67 years, education 10.77 ± 3.72 years). The AD, MCI, and NC groups showed progressively higher scores on ECOG (27.58 ± 10.27, 19.30 ± 7.50, 17.25 ± 5.93, P < 0.001), ADL (22.89 ± 4.36, 20.53 ± 2.07, 20.30 ± 2.27, P < 0.001), FAQ (4.88 ± 5.22, 1.07 ± 2.51, 0.49 ± 1.76, P < 0.001), and progressively lower scores on ACE III (56.18 ± 7.18, 69.49 ± 8.24, 81.34 ± 7.15, P < 0.001), MoCA-B (14.86 ± 3.64, 21.07 ± 3.50, 25.60 ± 2.70, P < 0.001), MMSE (21.07 ± 2.08, 26.2 ± 2.21, 27.96 ± 1.73, P < 0.001) (Table 1).

**Table 1:** Demographic and neuropsychological test information

	NC (n = 1008)	MCI (n = 633)	AD (n = 131)	Statistic	P value
Sex (male, %)	359, 35.6%	206, 32.5%	51, 38.9%	2.70	0.259
Age	65.12 ± 6.99	66.14 ± 6.78*	71.99 ± 6.67**#	60.67	< 0.001
Education years	12.21 ± 3.25	11.03 ± 3.21**	10.77 ± 3.72**	30.40	< 0.001
ECOG	17.25 ± 5.93	19.30 ± 7.50**	27.58 ± 10.27**#	74.07	< 0.001
ADL	20.30 ± 2.27	20.53 ± 2.07	22.89 ± 4.36**#	23.25	< 0.001
FAQ	0.49 ± 1.76	1.07 ± 2.51**	4.88 ± 5.22**#	56.05	< 0.001
ACE III	81.34 ± 7.15	69.49 ± 8.24**	56.18 ± 7.18**#	974.60	< 0.001
MoCA-B	25.60 ± 2.70	21.07 ± 3.50**	14.86 ± 3.64**#	810.69	< 0.001
MMSE	27.96 ± 1.73	26.2 ± 2.21**	21.07 ± 2.08**#	727.26	< 0.001
**DuCA-part 1**					
Verbal Fluence	1.55 ± 0.53	1.09 ± 0.53**	0.6 ± 0.55**#	265.07	< 0.001
Visual perception	2.56 ± 0.61	2.04 ± 0.8**	1.56 ± 0.92**#	152.33	< 0.001
Delay recall	2.84 ± 1.38	1.24 ± 1.34**	0.08 ± 0.35**#	1352.98	< 0.001
**Score of part 1**	6.95 ± 1.70	4.37 ± 1.75**	2.24 ± 1.09**#	1084.25	< 0.001
In male	6.88 ± 1.72	4.28 ± 1.69**	2.12 ± 1.26**#	350.14	< 0.001
In female	6.99 ± 1.69	4.41 ± 1.776**	2.31 ± 0.96**#	763.95	< 0.001
P value between sexes	0.297	0.361	0.319		
**DuCA part2**					
Category switching test	7.66 ± 1.69	5.80 ± 1.85**	3.82 ± 1.58**#	453.66	< 0.001
Picture memory	17.97 ± 2.83	14.62 ± 3.31**	7.95 ± 4.35**#	490.47	< 0.001
Auditory sentence memory	2.87 ± 1.68	0.94 ± 1.13**	0.12 ± 0.48**#	833.73	< 0.001
**DuCA-total score**	26.46 ± 4.01	18.41 ± 3.90**	10.13 ± 2.92**#	1978.51	< 0.001
In male	26.57 ± 4.23	18.15 ± 3.78**	9.77 ± 2.78**#	781.72	< 0.001
In female	26.4 ± 3.88	18.53 ± 3.95**	10.41 ± 2.95**#	1198.29	< 0.001
P value between sexes	0.538	0.251	0.216		

ECOG, Everyday Cognition. ADL, Activity of Daily Living Scale. FAQ, Functional Activity Questionnaire. ACE III, Addenbrooke’s Cognitive Examination III. MoCA-B, Montreal Cognitive Assessment Basic. MMSE, mini-mental state examination. DuCA, Dual-stage cognitive assessment. *, P < 0.05 compared with NC group. **, P < 0.001 compared with NC group. #, P < 0.001 compared with MCI group

Some participants in the NC group had subjective cognitive complaints. Some participants admitted that they had subjective cognitive complaints; those who presented to the cognitive clinic with subjective cognitive complaints were considered cognitively normal after neuropsychological testing. Thus, a relatively large proportion of the normative cohort had subjective cognitive complaints (n = 468; 46.4%). The underlying diseases in the NC group were as follows: 128 participants had coronary heart disease (12.7%), 355 had hypertension (35.2%), 203 had dyslipidemia (20.1%), 174 had diabetes (17.3%), and 193 had chronic respiratory disease (19.1%).

### Performance of NC and MCI groups in diagnostic assessments

The MCI group obtained lower scores than the NC group in the memory domain, including AVLT immediate recall, AVLT short delay-free recall, AVLT long delay-free recall, AVLT long delay-cued recall, AVLT recognition, BVMT immediate recall, BVMT short delay-free recall, BVMT long delay-free recall, and BVMT recognition (P < 0.001). The MCI group also obtained lower scores than the NC group in the language domain, including the BNT and VFT, and in the execution domain, including the STT-A and STT-B (P < 0.001) (Table 2).

**Table 2:** Comparison of diagnostic neuropsychological tests between NC group and MCI group

	NC (n = 1008)	MCI (n = 633)	P value	Effect size (95% CI)
AVLT immediate recall	17.38 ± 4.69	12.79 ± 3.80	< 0.001	1.05 (0.94 to 1.16)
AVLT short delay free recall	5.74 ± 2.33	3.03 ± 2.17	< 0.001	1.19 (1.08 to 1.30)
AVLT long delay free recall	5.23 ± 2.51	2.38 ± 2.11	< 0.001	1.20 (1.09 to 1.32)
AVLT long delay cued recall	5.18 ± 2.63	2.20 ± 2.04	< 0.001	1.23 (1.12 to 1.35)
AVLT recognition	21.53 ± 2.05	18.69 ± 3.07	< 0.001	1.14 (1.03 to 1.25)
BVMT immediate recall	20.86 ± 7.45	13.45 ± 7.19	< 0.001	1.01 (0.90 to 1.12)
BVMT short delay free recall	9.21 ± 2.68	6.22 ± 3.36	< 0.001	1.01 (0.90 to 1.12)
BVMT long delay free recall	9.18 ± 2.75	6.17 ± 3.44	< 0.001	0.99 (0.88 to 1.10)
BVMT recognition	11.59 ± 1.19	10.79 ± 1.94	< 0.001	0.53 (0.42 to 0.63)
BNT	24.27 ± 3.12	21.34 ± 3.92	< 0.001	0.85 (0.74 to 0.96)
VFT	17.07 ± 4.08	12.92 ± 3.56	< 0.001	1.07 (0.96 to 1.18)
STT-A	47.44 ± 15.30	59.69 ± 24.63	< 0.001	0.49 (0.39 to 0.59)
STT-B	128.27 ± 62.18	161.71 ± 54.18	< 0.001	-0.61 (-0.72 to -0.51)

AVLT, Auditory Verbal Learning Test. BNT, Boston Naming Test. BVMT, Brief Visuospatial Memory Test-Revised. VFT, Verbal Fluency Test. STT, Shape Trail Test

### Psychometric properties of DuCA

The NC, MCI, and AD groups had decreasing scores in DuCA-part 1 (6.95 ± 1.70, 4.37 ± 1.75, 2.24 ± 1.09, respectively, P < 0.001) and DuCA-total (26.46 ± 4.01, 18.41 ± 3.90, 10.13 ± 2.92, respectively, P < 0.001). We also analyzed whether sex affected DuCA scores. The results showed no differences in DuCA scores, including part 1 and total scores, between men and women (Table 1).

The correlation coefficient between DuCA-part 1 and DuCA-total was 0.84 (P < 0.001). The standardized Cronbach’s coefficient was 0.56 for the first part of the DuCA and 0.78 DuCA total. The inter-rater reliability and test-retest consistency (at an interval of 2 weeks) of the first part of the DuCA were 0.97 and 0.92 respectively. The inter-rater reliability and test-retest consistency of the DuCA total were 0.98 and 0.95 respectively. The correlation coefficients of DuCA-part 1 with ACE-III and MoCA were 0.66 (P < 0.001) and 0.85 (P < 0.001), respectively. The correlation coefficients of DuCA-total with ACE-III and MoCA were 0.78 (P < 0.001) and 0.83 (P < 0.001), respectively. There were weak correlations between years of education, DuCA Part 1 (r = 0.145, P < 0.001), and DuCA total score (r = 0.262, P < 0.001). In the NC group, only 43 participants (4.3%) obtained the maximum DuCA-part 1 score, and a single participant (0.1%) obtained the maximum DuCA-total score, indicating no obvious ceiling effect. Among individuals with MCI or AD, only 14 (1.8%) obtained a DuCA-part 1 score of 0, and none of the participants obtained a DuCA-total score of 0, indicating no obvious floor effect.

### Ability of the different scales to discriminate MCI

The discrimination ability was summarized using the AUC. DuCA-part 1 showed a discrimination ability (AUC = 0.87, 95% confidence interval [CI] 0.848–0.883) similar to that of ACE III (AUC = 0.86, 95%CI 0.838–0.874) and MoCA-B (AUC = 0.85, 95%CI 0.830–0.868). DuCA-total showed a higher AUC (0.93, 95%CI: 0.917–0.942) (Table 3).

**Table 3:** Ability to discriminate MCI from NC

	Estimate	SE	P value	OR (95% CI)	BIC	Accuracy	AUC
ACE III	-0.20	0.010	< 0.001	0.82 (0.80 to 0.83)	1476	0.79	0.87 (0.848 to 0.883)
MoCA-B	-0.44	0.023	< 0.001	0.65 (0.62 to 0.68)	1581	0.77	0.86 (0.838 to 0.874)
ADL	0.05	0.028	0.056	1.06 (1.0 to 1.12)	2198	0.62	0.54 (0.508 to 0.566)
FAQ	0.16	0.029	< 0.001	1.18 (1.11 to 1.25)	2165	0.63	0.57 (0.546 to 0.604)
DuCA-part 1	-0.79	0.039	< 0.001	0.45 (0.42 to 0.49)	1541	0.79	0.85 (0.830 to 0.868)
DuCA-total	-0.53	0.026	< 0.001	0.59 (0.56 to 0.62)	1112	0.87	0.93 (0.917 to 0.942)

ACE III, Addenbrooke’s Cognitive Examination III. MoCA-B, Montreal Cognitive Assessment Basic. ADL, Activity of Daily Living Scale. FAQ, Functional Activity Questionnaire. DuCA, Dual-stage cognitive assessment. BIC, Bayesian information criterion

### Discrimination ability of DuCA for MCI

To validate the efficacy of DuCA in populations with different levels of education, we stratified the participants according to education. Based on years of education, we divided the participants of the MCI and NC groups into four education levels: ≤6 years of education (n = 108); > 6 years of education and ≤ 99 years (n = 347); > 9 years to ≤ 12 years (n = 569); > 12 years of education (n = 617). For each education level, sex, age, and years of education between the MCI and NC groups were compared, and no significant differences were found. The AUC for discriminating MCI from the NC of DuCA-part 1 and DuCA-total was calculated for each group. At different education levels, the AUC of DuCA-part 1 ranged from 0.83 to 0.84, and that of DuCA-total ranged from 0.89 to 0.94. We provided the recommended cutoff values and corresponding sensitivities and specificities for each education level (Table 4).

**Table 4:** Discrimination ability of DuCA

				Discrimination ability for MCI						
**MCI from NC**	NC	MCI	P value		AUC	Cut-off	Sensitivity	Specificity	PPV*	NPV*
**Education** ≤ **6 years**	n = 55	n = 53		DuCA-1	0.83	≤ 5	90.6%	54.6%	66.6%	85.3%
Sex (male, %)	13, 23.6%	12, 22.6%	0.902			≤ 3	62.3%	87.3%	83.1%	69.8%
Age	65.27 ± 6.73	67.26 ± 7.2	0.140	DuCA-T	0.89	≤ 20	88.7%	74.6%	77.7%	86.8%
Education years	5.08 ± 1.31	4.78 ± 1.3	0.237			≤ 17.5	73.6%	87.3%	85.3%	76.8%
**Education > 6–9 years**	n = 178	n = 169		DuCA-1	0.86	≤ 6	90.5%	60.1%	69.4%	86.4%
Sex (male, %)	56, 31.5%	52, 30.8%	0.889			≤ 4	62.1%	88.8%	84.7%	70.1%
Age	65.76 ± 5.55	66.55 ± 6.10	0.21	DuCA-T	0.93	≤ 22	92.3%	83.7%	85.0%	91.6%
Education years	8.76 ± 0.51	8.70 ± 0.62	0.277			≤ 21	84.0%	89.3%	88.7%	84.8%
**Education > 9–12 years**	n = 329	n = 240		DuCA-1	0.85	≤ 6	86.7%	66.3%	72.0%	83.3%
Sex (male, %)	108, 32.8%	70, 29.2%	0.352			≤ 4	52.5%	94.5%	90.5%	66.5%
Age	64.50 ± 6.28	65.37 ± 6.21	0.102	DuCA-T	0.93	≤ 23	89.6%	85.4%	86.0%	89.1%
Education years	11.36 ± 0.77	11.25 ± 0.83	0.116			≤ 22.5	85.8%	90.0%	89.6%	86.4%
**Education > 12 years**	n = 446	n = 171		DuCA-1	0.84	≤ 6	81.9%	69.7%	73.0%	79.4%
Sex (male, %)	182, 40.8%	72, 42.1%	0.769			≤ 5	67.8%	85.4%	82.3%	72.6%
Age	65.31 ± 7.95	66.46 ± 7.92	0.109	DuCA-T	0.94	≤ 23	91.2%	87.0%	87.5%	90.8%
Education years	15.1 ± 1.81	14.96 ± 1.78	0.385			≤ 22	86.0%	90.8%	90.3%	86.6%
**Total**				DuCA-1	0.85	≤ 6	87.1%	65.1%	71.4%	83.5%
						≤ 5	73.9%	81.5%	80.0%	75.7%
				DuCA-T	0.93	≤ 22.5	89.6%	85.1%	85.7%	89.1%
**AD from MCI**	MCI	AD		DuCA-1	0.84	≤ 3	91.6%	66.2%	73.0%	88.7%
						≤ 2	53.4%	86.7%	80.1%	65.0%
	n = 633	n = 131		DuCA-T	0.95	≤ 15	98.5%	80.0%	83.1%	98.2%
						≤ 13	85.5%	90.8%	90.3%	86.2%

DuCA, Dual-stage cognitive assessment. DuCA-1, DuCA-part 1. DuCA-T, DuCA-total. PPV, positive predictive value. NPV, negative predictive value. *, based on a 50% prevalence

### Discrimination ability of DuCA for AD

We calculated the ability of the DuCA to discriminate between AD and MCI. The DuCA-Part 1 had an AUC of 0.84. When the cutoff was set to ≤ 3, the sensitivity and specificity were 91.6% and 66.2%, respectively. When the cutoff was set to ≤ 2, the sensitivity and specificity were 53.4% and 86.7%, respectively. The DuCA-total had an AUC of 0.93. When the cutoff was set to ≤ 15, the sensitivity and specificity were 98.5% and 80.0%, respectively. When the cutoff was set to ≤ 13, the sensitivity and specificity were 85.5% and 90.8%, respectively (Table 4).

### Discrimination ability for auditory and visual memory impaired MCI

Based on the presence of impaired auditory (AVLT) or visual (BVMT) memory indicators, the MCI group was further divided into MCI with auditory impairment (MCI-A, n = 221, 34.9%), MCI with auditory and visual impairment (MCI-AV, n = 324, 51.2%), and other groups (n = 88, 13.9%).

For MCI-A, DuCA-part 1 (AUC = 0.80, 0.767–0.830) had a similar discrimination ability to ACE III (AUC = 0.82, 0.793–0.850) and MoCA-B (AUC = 0.80, 0.775–0.835) and higher than MMSE (AUC = 0.69, 0.647–0.723). For MCI-AV, DuCA-Part 1 had a similar discrimination ability (AUC = 0.91, 0.888–0.923) to ACE III (AUC = 0.90, 0.882–0.920) and MoCA-B (AUC = 0.91, 0.891–0.926) and higher than MMSE (AUC = 0.80, 0.772–0.828). For other MCI, DuCA-part 1 had a relatively lower discrimination ability (AUC = 0.77, 0.719–0.814) than ACE III (AUC = 0.84, 0.806–0.882), similar to MoCA-B (AUC = 0.79, 0.748–0.835) and higher than MMSE (AUC = 0.66, 0.603–0.723).

Compared to the other tools, DuCA-total had better discrimination ability (AUC for MCI-A:0.89, 0.871–0.913; AUC for MCI-AV:0.96, 0.951–0.970; and AUC for other MCI:0.91, 0.879–0.937) (Table 5).

**Table 5:** Discrimination ability in MCI subgroups

	MCI-A (n = 221)	MCI-AV (n = 324)	MCI-other (n = 88)
ACE III (AUC)	0.82 (0.793 to 0.850)	0.90 (0.882 to 0.920)	0.84 (0.806 to 0.882)
MoCA-B (AUC)	0.80 (0.775 to 0.835)	0.91 (0.891 to 0.926)	0.79 (0.748 to 0.835)
MMSE (AUC)	0.69 (0.647 to 0.723)	0.80 (0.772 to 0.828)	0.66 (0.603 to 0.723)
DuCA-part 1 (AUC)	0.80 (0.767 to 0.830)	0.91 (0.888 to 0.923)	0.77 (0.719 to 0.814)
DuCA-total (AUC)	0.89 (0.871 to 0.913)	0.96 (0.951 to 0.970)	0.91 (0.879 to 0.937)

MCI-A, MCI with auditory impairment. MCI-AV, MCI with auditory and visual impairment. ACE III, Addenbrooke’s Cognitive Examination III. MoCA-B, Montreal Cognitive Assessment Basic. MMSE, mini-mental state examination. DuCA, Dual-stage cognitive assessment

## Discussion

The DuCA is a two-stage neuropsychological test with screening capabilities similar to those of commonly used cognitive assessment tools for MCI and AD. The concise and flexible features of DuCA allow it to be used as a quick or complete screen, depending on the scenario and purpose. The best and worst cognitive populations can be identified in Part 1, which requires only 3 min. Memory impairment is the earliest typical cognitive impairment in AD. DUCA has a high memory component and is efficient in assessing memory function. DuCA can detect different memory components and help identify abnormalities in the corresponding pathological processes.

Some brief tests that consist of only a few items, such as the Mini-Cog, have shown similar performance in detecting dementia as traditional detection tools [29]. However, most of these brief assessment tools still do not adequately distinguish between MCI and normal cognition [30]. Some brief tools, such as the Rapid Cognitive Screen [31], have also shown a rapid screening ability for MCI [32]. However, other time-consuming assessment tools are required when further assessment of possibly positive individuals is required. Therefore, we aimed to develop a brief tool that included two parts. The first part is used for rapid screening, and the other part is performed when necessary, which, in combination with the first part, can increase the accuracy of the test.

Several screening scales are currently available for large-scale implementation in China. The advantage of the DuCA over these tools is the flexibility of its two-stage approach. The first part of the DuCA is close to the ACE III and MoCA in its ability to discriminate between MCI and NC, but it takes less time. Some tools for evaluating daily abilities, such as ADL and FAQ, are short and easy to use. However, based on the AUC values, the first part of the DuCA was more discriminative for MCI than for ADL or FAQ. When the full version of the DuCA was used, its ability to distinguish MCI from NC was further improved, especially in terms of specificity. By strategically using the two parts of the DuCA, individuals with cognitive impairment can be screened accurately in a relatively short period. Time-saving particularly advantageous for large-scale screening.

Grober et al. experimented with a two-step approach to screen for dementia in primary care with encouraging results [33]. They used the Memory Impairment Screen (four words), animal fluency, and reciting months backward in stage 1, and immediate recall of the free and cued selective reminders test (picture version) in stage 2. In DuCA, the first stage includes semantic executive, delayed recall, and visuospatial ability. The second stage comprises auditory, visual, and semantic executive abilities. Thus, more emphasis has been placed on testing memory functions while retaining language, executive, and visuospatial functions. This is reflected in the fact that DuCA Part 2 contains both auditory and visual memory functions.

According to a meta-analysis, memory tests are the most effective items for screening for MCI in primary care, with delayed recall showing better results than immediate recall [34]. The objects and psychological processes of auditory and visual memory are different, and some studies have suggested that visual memory performance is superior to that of auditory memory [35, 36]. Some researchers believe that auditory and visual memory are distinct memory systems because of different neural circuits [37, 38]. For older adults, visual memory of pictures seems to be more advantageous than that of words [39]. Patients with MCI may have impaired visual memory [40] and tests for visual memory have long been validated to distinguish individuals with MCI from NC individuals [41]. Diminished visual memory is associated with hippocampal atrophy [42]. Hippocampal subregion atrophy is characteristically associated with visual memory impairment in individuals with subjective cognitive decline and MCI [43]. Visual short-term memory performance is closely associated with tau in the entorhinal cortex and inferior temporal lobe. Even in asymptomatic carriers, it is closely associated with amyloid and can therefore be regarded as an early marker of AD pathology [44, 45]. Visual memory evaluation using computerized visual memory assessment with the Cambridge Neuropsychological Test Automated Battery (CANTAB) can distinguish between different types of MCI and predict changes in cognitive status [46]. A visual reproduction test using the Wechsler Memory Scale-Revised can predict the conversion from MCI to AD [47]. Spatial delayed recall, rather than auditory memory items, predicts rapid conversion from cognitively normal to MCI [48]. Therefore, it is necessary to include visual memory detection items in the assessment tool.

The DuCA is a comprehensive multidomain assessment tool. It includes an evaluation of verbal, executive, and visuospatial functions, and a comprehensive memory function assessment. Even though it is brief, it still achieves similar or even better results than commonly used traditional scales. Notably, the performance of the MoCA and MMSE in our MCI cohort was consistent with the results of previous studies [34, 49]. Several studies support our results that multisensory integration is a suitable screening tool for older adults [50, 51]. Using the test your memory for mild cognitive impairment, a brief tool that combines verbal and visual memory assessment, can effectively identify patients with MCI and AD with minimal time and training, and can be combined with other brief assessment tools to further improve accuracy [51].

Education was correlated with baseline cognitive levels in older adults [52, 53]. The performance of DuCA varied among participants with different education levels; therefore, we recommend that different cutoff values based on education level be considered to obtain a more accurate identification.

Our results showed that the DuCA could discriminate between possible AD and MCI. When used for this purpose, the first part of the DuCA had good sensitivity (91.6%) and moderate specificity (66.2%). The DuCA total score significantly improved its specificity (80.0% or 90.8%). Using the first part, some individuals with possible AD may directly be screened; other suspected individuals undergo the second part. This modest reduction in time may be important when a large number of candidates are screened. Therefore, it is considered a good screening tool.

Some participants with MCI only had auditory memory impairment. However, our study showed that using the DuCA did not reduce the ability to discriminate between this population. According to the AUC and 95% CI, the DuCA performed similar to the ACE III and MoCA-B for participants with auditory memory impairment alone and MCI with both auditory and visual memory impairment in terms of their ability to distinguish them from NC. When the DuCA total score was used, the distinguishing ability was significantly improved.

### Limitations

This study had some limitations. The lack of testing for biological markers makes it impossible to explain the correlation between DuCA and pathological changes that lead to cognitive decline. Hence, it was impossible to assess whether DuCA could discriminate between cognitive decline caused by different etiologies. The design that focuses primarily on the assessment of memory function may reduce the sensitivity of DuCA to other etiologies. DuCA may be more appropriate for detecting early stage AD, but has the disadvantage of missing atypical cases with less involvement of memory and executive function. The ability to detect vascular cognitive impairment could not be determined because patients with a history of stroke were not enrolled. The predictive power of DuCA for outcomes could not be evaluated because of the need for more data from longitudinal follow-up. Due to the limitation of the source of participants in this study, the results may differ from the overall situation in a larger region. When using the scale in other areas, especially those with different cultural backgrounds and educational levels, it should be revalidated, and a suitable cutoff should be found first. Our normative cohort that included participants were from the community and a cognitive clinic consisted of individuals with subjective cognitive complaints also. Therefore, there may be subtle differences between the data from our normative group and that from a fully healthy population, which may have resulted in reduced sensitivity.

## Conclusion

The DuCA is a two-stage brief assessment tool that is well suited to distinguish adults with cognitive impairment from the NC population and has excellent results for MCI. The first part of the DuCA can be used for rapid screening and is supplemented by the second part for individuals with suspected positive results to increase diagnostic reliability. DuCA can be used for large-scale cognitive screening in primary care settings, saving the time required for cognitive assessment and eliminating the need for extensive training of assessors.

## Electronic supplementary material

Below is the link to the electronic supplementary material.

Supplementary Material 1 Table S1 Dual-stage cognitive assessment (DuCA)

## Data Availability

The datasets used and analyzed during the current study are available from the corresponding author on reasonable request.
