# Supplementary material for: Dual-stage cognitive assessment: a two-stage screening for cognitive impairment in primary care
Source: BMC Psychiatry. 2023 May 25;23:368. doi: 10.1186/s12888-023-04883-w (PMC10210296; doi:10.1186/s12888-023-04883-w)
Supplement: Supplementary file 1 — Supplementary Material 1 Table S1 Dual-stage cognitive assessment (DuCA) [file 12888_2023_4883_MOESM1_ESM.docx]

**Table S1 Dual-stage cognitive assessment (DuCA)**

| **Part1** | Content | Score |
| --- | --- | --- |
| Prospective memory | Please remember that after completing all the questions, draw a triangle in the bottom right corner and write your name. |  |
| Immediate recall | Five items: Rose, Foot, Sofa, Blue, and Knife.  The subject needs to finish the second trial even if all the words have been recalled in the first trial. | 5 |
| **Verbal fluency** | Naming as many fruits as possible in one minute.  Scoring:  2 points are given if the subject names more than 13 fruits in 1 minute.  1 point is given if the subject names more 8-12 fruits in 1 minute.  No point is given if the subject names less than 7 fruits in 1 minute. | 2 |
| **Visual perception** | The examiner points to the picture in the visual perception part and tells the subject "Now please look at this picture. There are many overlapped objects in this picture. Name the objects that you can distinguish. "  Scoring: There are 10 items in the picture: scissors, a cup, a T-shirt (shirt, underwear), a watch, a banana, a leaf (foliage), a table lampa , key, a candle and a spoon.  3 points are given if the subject identifies 9-10 items.  2 points are given if the subject identifies 6-8 items.  1 point is given if the subject identifies 4-5 items.  No point is given if the subject identifies 3 or less than 3 items. | 3 |
| Meta-memory | Guidance: "Of the words you were asked to memorize at the beginning, how many do you estimate you can recall now?"  Record the answered number. |  |
| **Delayed recall** | Points are given to correctly recalled words without any cue (1 point for each item). | 5 |
| Prospective memory | "The items are all done. Now do you have anything else to do?"  4 points are given when not reminded and completely correct.  3 points are given when write partially.  2 points are given when forget to write at the beginning and write correctly after reminding (what to write in the bottom right corner).  1 point given when write partially after the reminder.  No point is given when can't write at all after reminder. | 4 |
| **Part2** |  |  |
| Picture naming | Name the 12 pictures presented. | 12 |
| Visual immediate recall 1 | Observe the pictures for 30 seconds, move them away and recall immediately (1 point for each item). | 12 |
| Visual immediate recall 2 | Observe the pictures for 1 minute more. Move them away and recall immediately (1 point for each item). | 12 |
| **Auditory sentence memory** | Give one point for each item accurately stated. Give 6 points for ≥6 items. | 6 |
| **Category switching test** | Orally generate objects in the order of alternating animals and fruits within 60 seconds, such as dog, apple, horse, orange, mouse, banana, etc.  Scoring: each correctly stated item is given 0.5 points out of 10. | 10 |
| Meta-memory | Guidance: "Of the pictures you were asked to memorize at the beginning, how many do you estimate you can recall now?"  Record the answered number. |  |
| Visual delayed free recall | Points are given to correctly recalled pictures without any cue (1 point for each item). | 12 |
| **Visual memory** | Total score of visual immediate recall 2 and visual delay free recall divided by 2. | 12 |
| Visual delayed recognition | Points are given to correctly recalled pictures with cue (1 point for each item). | 12 |
| Auditory Sentence delayed free recall | Give one point for each item accurately stated. Give 6 points for ≥6 items. | 6 |
| Auditory Sentence recognition | Give one point for each item accurately recognited. Give 6 points for ≥6 items. | 6 |

Bolded font indicates items that count in the total score.
